# The Complete Genome and Phenome of a Community-Acquired *Acinetobacter baumannii*

**DOI:** 10.1371/journal.pone.0058628

**Published:** 2013-03-19

**Authors:** Daniel N. Farrugia, Liam D. H. Elbourne, Karl A. Hassan, Bart A. Eijkelkamp, Sasha G. Tetu, Melissa H. Brown, Bhumika S. Shah, Anton Y. Peleg, Bridget C. Mabbutt, Ian T. Paulsen

**Affiliations:** 1 Department of Chemistry and Biomolecular Sciences, Macquarie University, Sydney, New South Wales, Australia; 2 School of Biological Sciences, Flinders University, Adelaide, South Australia, Australia; 3 Department of Microbiology, Monash University, Clayton, Victoria, Australia; 4 Department of Infectious Diseases, The Alfred Hospital, Melbourne, Victoria, Australia; University of Florida, United States of America

## Abstract

Many sequenced strains of *Acinetobacter baumannii* are established nosocomial pathogens capable of resistance to multiple antimicrobials. Community-acquired *A. baumannii* in contrast, comprise a minor proportion of all *A. baumannii* infections and are highly susceptible to antimicrobial treatment. However, these infections also present acute clinical manifestations associated with high reported rates of mortality. We report the complete 3.70 Mbp genome of *A. baumannii* D1279779, previously isolated from the bacteraemic infection of an Indigenous Australian; this strain represents the first community-acquired *A. baumannii* to be sequenced. Comparative analysis of currently published *A. baumannii* genomes identified twenty-four accessory gene clusters present in D1279779. These accessory elements were predicted to encode a range of functions including polysaccharide biosynthesis, type I DNA restriction-modification, and the metabolism of novel carbonaceous and nitrogenous compounds. Conversely, twenty genomic regions present in previously sequenced *A. baumannii* strains were absent in D1279779, including gene clusters involved in the catabolism of 4-hydroxybenzoate and glucarate, and the *A. baumannii* antibiotic resistance island, known to bestow resistance to multiple antimicrobials in nosocomial strains. Phenomic analysis utilising the Biolog Phenotype Microarray system indicated that *A. baumannii* D1279779 can utilise a broader range of carbon and nitrogen sources than international clone I and clone II nosocomial isolates. However, D1279779 was more sensitive to antimicrobial compounds, particularly beta-lactams, tetracyclines and sulphonamides. The combined genomic and phenomic analyses have provided insight into the features distinguishing *A. baumannii* isolated from community-acquired and nosocomial infections.

## Introduction

*Acinetobacter baumannii* is a significant nosocomial pathogen [1], known for its high intrinsic and laterally acquired resistance to antimicrobials [2], [3] as well as its persistence on various abiotic surfaces [4]-[6]. The complete genome sequences of ten *A. baumannii* strains have been determined to date: 1656-2 [7], AB0057 [8], AB307-0294 [8], ACICU [9], ATCC 17978 [10], AYE [11], MDR-TJ [12], MDR-ZJ06 [13], SDF [11] and TCDC-AB0715 [14]. Nine of these are nosocomial isolates, whereas *A. baumannii* SDF was isolated from a human body louse [11]. These genome sequences have demonstrated extensive divergence due to the acquisition and accretion of various mobile genetic elements, particularly those contributing to antimicrobial resistance [15]. One mobile element of clinical import is the *A. baumannii* antibiotic resistance island (AbaR), that encodes resistance to a multitude of antibiotics and heavy metals [15].

Research regarding *A. baumannii* has occurred primarily within the context of the clinical milieu, with little known about potential environmental reservoirs of this organism. Several non-nosocomial niches of *A. baumannii* have been identified, including human lice [16], [17], hydrocarbon contaminated soils [18], [19], the plant rhizosphere [20], [21] and estuaries [22], [23]. *A. baumannii* is also known to exist outside the hospital environment as a commensal of the skin [24] and nasopharynx [25] of humans. This organism is also a public health issue outside of the hospital setting, in the form of community-acquired *A. baumannii* (CA-AB) infections.

Infections caused by CA-AB are clinically and epidemiologically distinct from their nosocomial counterparts [26]. CA-AB infections are uncommon and highly fatal, comprising less than 10% of all *A. baumannii* infections [27], [28] but resulting in mortalities ranging from 30–62% [27]–[30]. These infections are also antimicrobial susceptible [26], [30] and present a more acute clinical manifestation [30], but are thought not to be reservoirs of nosocomial outbreaks [26].

The majority of CA-AB infections occur in individuals with underlying comorbidities, who reside in tropical and subtropical climates [28]. Incidences of CA-AB infection have been reported within various regions of the Asia Pacific such as Taiwan [31], Hong-Kong [30], Singapore [32], Korea [33] and Australia [34]. To a lesser extent, CA-AB infections have also been observed in non-tropical regions [27] and in otherwise healthy children and adults [35]–[39]. Indigenous Australians in the Northern Territory are overrepresented relative to the general population in rates of community-acquired bacteraemic pneumonia caused by *A. baumannii* and other pathogens [29], [34]. This disparity has been attributed to the interaction of both monsoonal climate and a high prevalence of comorbidities in the indigenous Australian population including alcoholism, diabetes mellitus, chronic obstructive pulmonary disease and cigarette smoking [25], [34], [40].

To explore the underlying basis of epidemiological and phenotypic differences between nosocomial and community-acquired strains of *A. baumannii*, we determined the complete genome sequence of the CA-AB isolate D1279779 and phenotypically profiled this strain using phenotype microarrays. The genome and phenome of D1279779 was subsequently compared to completely sequenced nosocomial *A. baumannii* strains.

## Materials and Methods

### Bacterial strains, culture conditions and genomic DNA extraction

The *A. baumannii* strain D1279779 was kindly provided by the Menzies School of Health Research (Darwin, Australia). This strain was isolated in 2009 from a bacteraemic infection of an indigenous Australian male at the Royal Darwin Hospital, where the identity and antimicrobial susceptibilities of this isolate were previously determined with a VITEK 2 System (bioMérieux). *A. baumannii* D1279779 was cultured in lysogeny broth (LB) and lysogeny broth agar (LBA) (both without glucose) at 37 °C. Genomic DNA was extracted with the Wizard Genomic DNA Purification Kit (Promega) from 1 mL of overnight culture as per the manufacturer’s protocol.

### DNA sequencing, genome assembly and annotation

*A. baumannii* D1279779 genomic DNA was prepared and sequenced by 454 FLX pyrosequencing (Roche Diagnostics) at the Ramaciotti Centre for Gene Function Analysis (University of New South Wales, Sydney). The sequences reads were assembled *de novo* with MIRA [41] using the default parameters. The ninety-four contiguous sequences of D1279779 were reordered relative to the ten currently complete genomes of *A. baumannii* using MAUVE [42]. The highest level of synteny was observed with the *A. baumannii* ACICU genome [9], which was subsequently utilised as a reference in Projector2 [43] to design oligonucleotides for gap closure by PCR and sequencing. Amplicons for gap closure were generated using AccuPrime *Pfx* Mastermix (Invitrogen) or GoTaq DNA Polymerase (Promega) as per the manufacturer’s protocol, with variations in the annealing temperature (45 °C to 65 °C) and extension time (2 to 12 min) according to estimated gap sizes. The resultant amplicons were purified using the QIAquick PCR Purification Kit (Qiagen) and cloned into pGEM-T Easy (Promega), or directly sequenced using bidirectional dideoxysequencing performed by the Macquarie University DNA Analysis Facility (Sydney, Australia). The resultant chromatograms were edited and assembled in ChromasPro **(**Technelysium Pty. Ltd.) and crosschecked against the D1279779 genome by use of the BLASTN [44] application integrated into BioEdit [45]. The presence and directionality of the six rRNA operons in the genome was confirmed by amplification of the gap and sequencing of small junctions flanking this region. The genome assembly was finalised with the aid of CLC Sequence Viewer 6 (CLCbio). Genome annotation was conducted with the RAST automated annotation engine [46] and manual curation was performed with the aid of Artemis [47]. UGENE was routinely utilised for genome browsing and analysis [48]. The nucleotide sequences of the D1279779 chromosome and the plasmid pD1279779 have been deposited into GenBank with the accession numbers CP003967 and CP003968 respectively.

### Comparative genomics and accessory element identification

*A. baumannii* D1279779 was compared to the ten published *A. baumannii* genomes by a reciprocal BLASTP [49] search to identify putative orthologs at an e-value cutoff of 10^-5^. Trinucleotide composition of the DNA sequence was computed by χ^2^ analysis using a 2000 bp sliding window with a 1000 bp overlap [50]; regions containing χ^2^ values >500 were suggestive of atypical trinucleotide composition. Evidence derived from BLASTP and χ^2^ analysis was used to identify regions of genomic plasticity (RGPs) [51], [52], defined as either any putative mobile genetic element or contiguous cluster of genes present in D1279779 and three or less strains. Genes clusters absent in D1279779 but present in more than three other strains were also considered RGPs. The identity of insertion sequences present in the D1279779 genome were elucidated with BLASTP searches conducted in ISFinder [53].

The phylogenetic relationship of D1279779 to sequenced *A. baumannii* strains was inferred with a bootstrapped neighbour-joining analysis in MEGA5 [54] based on the concatenated nucleotide sequences of six of the seven reference genes utilised in the multilocus sequence typing (MLST) of *A. baumannii* [55]. The *fusA* gene was excluded from this analysis as it displayed atypical trinucleotide composition in D1279779, suggesting potential lateral acquisition of this gene.

### Phenotype microarray testing

The phenomes of *A. baumannii* D1279779 and three nosocomial strains, ACICU, ATCC 17978 and AYE, were assayed with the Biolog Phenotype MicroArray™ (PM) system [56] to identify compounds that could serve as sole carbon (PM1-2; 190 compounds) or nitrogen sources (PM3; 95 compounds). Additionally, sensitivities to stress conditions (PM9-10; 192 conditions) and various antimicrobials compounds (PM11-20; 240 antimicrobials) were also investigated. All phenotypic tests were performed as per the manufacturer’s protocol, except cryogenic stocks of *A. baumannii* were streaked onto either LBA medium (PM1-2 and PM9-20) or Reasoner’s 2A agar (Difco) (PM3). The bacterial suspension for nitrogen source testing was supplemented with D-xylose as the sole carbon source at a concentration of 20 mM. Following inoculation, all PM plates were incubated in an OmniLog reader (Biolog) aerobically at 37 °C for 48 h. Reduction of the tetrazolium-based dye (colourless) to formazan (violet) was monitored and recorded at 15 min intervals by an integrated charge-coupled device camera. The resultant data were analysed with the supplied manufacturer’s software, resulting in a time-course curve for colorimetric change equating to respiration rate. The phenotypes were classified on the basis of the maximal curve height; a phenotype was considered positive if the height was greater than 115 and 101 OmniLog units for nitrogen sources and all other phenotypes, respectively. Data that exceeded these cutoff values as the result of colouration from certain compounds was excluded from analysis. Observed phenotypic differences between the strains were linked to differences in genotype through a combined analysis of the EcoCyc [57], MetaCyc [58], and KEGG [59] metabolic databases and additional literature searching.

### Independent confirmatory testing of phenotype microarray data

Five millilitres of M9 minimal media (Sigma-Aldrich), supplemented with varied carbon source compounds (20 mM), was inoculated with a single colony of *A. baumannii* D1279779 or ACICU previously streaked on LBA medium. Cultures were incubated with shaking at 37 °C for 24 h, with the observation of turbidity from cellular replication deemed to signify a positive phenotype. A minimum of three temporally distinct replicates were performed for each tested compound, in addition to substrate and inoculation negative controls. The carbon sources tested were: L-arabinose, bromosuccinic acid, (±)-carnitine hydrochloride, L-carnitine hydrochloride, disodium fumarate, α-D-glucose, L-histidine monohydrochloride monohydrate, polysorbate 80, putrescine dihydrochloride, L-pyroglutamic acid, quinic acid, sodium 4-hydroxybenzoate, sodium acetate trihydrate, trisodium citrate dihydrate, sodium D-gluconate, and D-xylose. All chemicals were sourced from Sigma-Aldrich (purity ≥96%), dissolved in sterile distilled water and filter-sterilised. Phenotypic testing of α-D-glucose and D-gluconic acid utilisation was further conducted in inoculation fluid zero (IF-0) [56] (without sodium pyruvate and tetrazolium dye). Further experiments with α-D-glucose were supplemented with 10 µM methoxatin disodium salt (pyrroloquinoline quinone) with the corresponding negative control.

## Results and Discussion

### Genomic features

**Figure 1:**
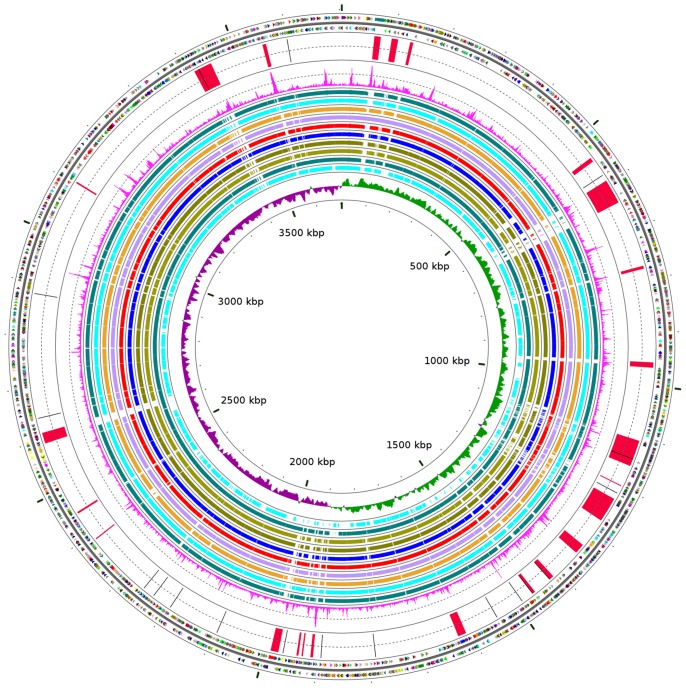
Genome map of *Acinetobacter baumannii* D1279779. The two outermost circles denote positions of protein coding sequences (CDSs) on the positive (circle 1) and negative (circle 2) strands coloured according to clusters of orthologous groups (COGs) [99] functional category: A (lavender), B (apricot), C (olive), D (light brown), E (dark green), F (electric pink), G (electric green), H (peach), I (red), J (dark red), K (midnight blue), L (plum), M (teal), N (blue), O (aquamarine), P (orange), Q (yellow), R (dark grey), S (grey), T (light purple), U (light green), V (light yellow), and unknown COG (black). Circle 3 represents positions of identified regions of genomic plasticity (red) and ISAb13s (black) ordered clockwise from the origin of chromosomal replication as outlined in Table 2 and Table 4, respectively. Circle 4 denotes the calculated chi-squared values based on the trinucleotide composition of the DNA sequence. Circles 5-14 show DNA conservation between D1279779 and other sequenced *A. baumannii* strains based on pairwise BLASTN alignments (evalue threshold 1e-10). Strain comparisons (outermost to innermost): 1656-2 (teal), ACICU (aquamarine), MDR-ZJ06 (orange), MDR-TJ (light purple), TCDC-AB0715 (red), AB307-0294 (blue), AB0057 (olive), AYE (green-brown), ATCC 17978 (teal) and SDF (aquamarine). The innermost circle denotes positive (green) and negative (purple) GC-skew and the scale in kilobase pairs. The CGView software [100] was utilised to construct the genome map.

The complete genome of *A. baumannii* D1279779 was determined and found to consist of a 3704285 bp circular chromosome and a plasmid of 7416 bp, dubbed pD1279779. A total of 3479 genes were annotated on the chromosome including 65 tRNAs, 6 rRNA operons and 3388 predicted protein coding sequences (CDS) (Table 1, Figure 1), which included 1019 annotated CDS (30%) predicted to encode hypothetical proteins. The plasmid pD1279779, unlike many previously sequenced *A. baumannii* plasmids, does not encode any insertion sequences or genes involved in antimicrobial resistance [60]. Plasmid pD1279779 appears to be of mosaic origin, with a replication *repA* gene sharing 100% nucleotide sequence identity with the *A. baumannii* plasmid p203 [60] and a 2609 bp segment sharing 99% nucleotide identity with a region from the otherwise unrelated organic peroxide resistance plasmid pMAC of *A. baumannii* ATCC 19606 [61].

**Table 1:** Comparative genome features of *Acinetobacter baumannii* D1279779.

Strain	D1279779	ATCC 17978	ACICU	AYE
Size (base pairs)	3704285	3976747	3904116	3936291
Plasmids	1	2	2	4
G+C content (%)	39.00	38.94	39.03	39.40
Protein-coding sequences (CDSs)	3388	3787	3670	3607
Insertion sequences	18	14	14	33
Average gene length	935	888	929	951
Coding regions (%)	85.60	84.50	84.87	86.13
rRNA operons	6	5	6	6
tRNAs	65	69	64	72

### The phylogeny and synteny of *A. baumannii*

**Figure 2:**
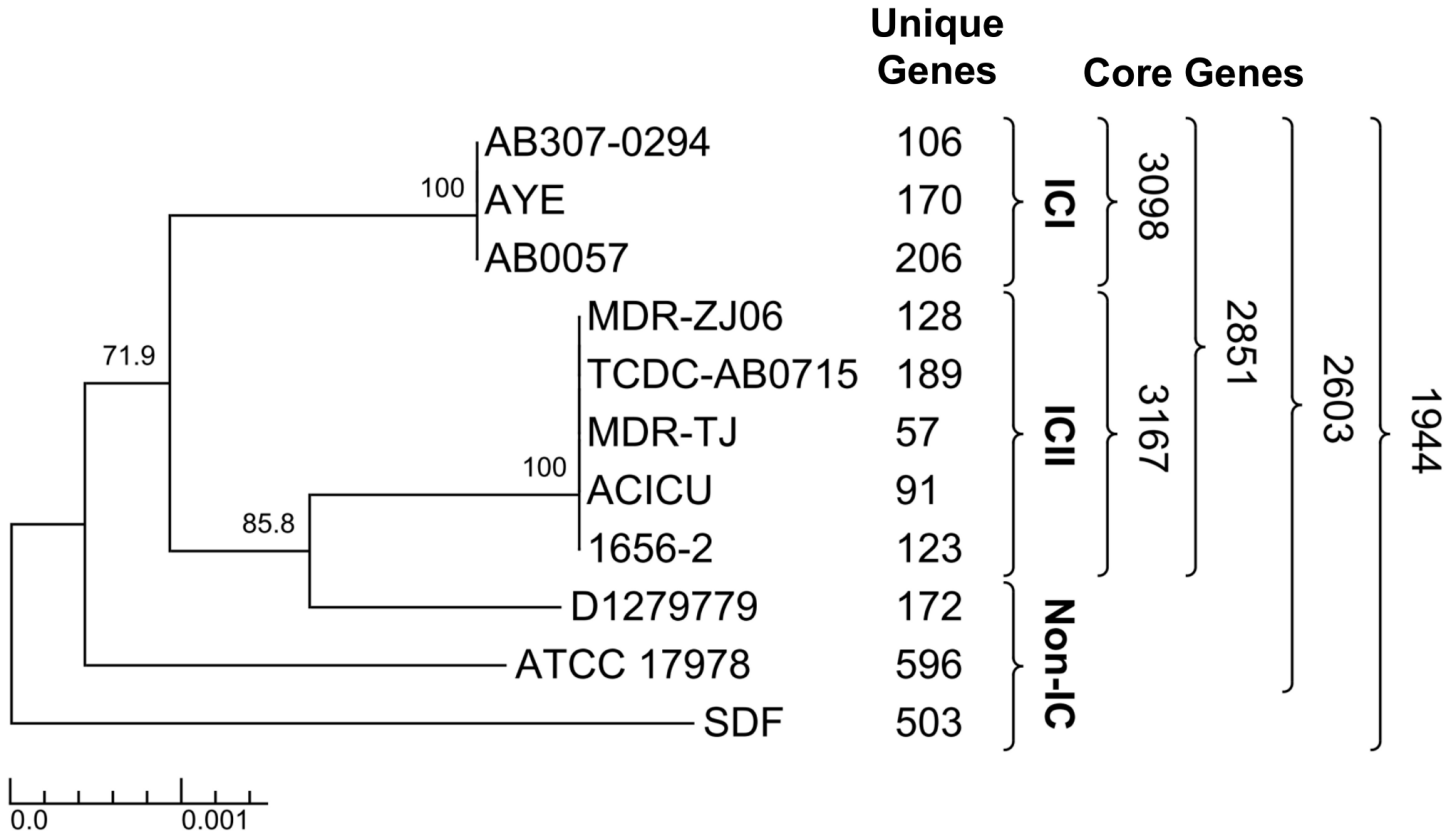
Phylogenetic lineage of *Acinetobacter baumannii*. The phylogenetic relationship of all completely sequenced *A. baumannii* strains was inferred by the neighbour-joining method conducted in MEGA5 [54] using concatenated nucleotide sequences of six reference genes based on the multilocus sequence tag scheme of *A. baumannii* (*cpn60*, *gltA*, *pyrG*, *recA*, *rplB* and *rpoB*). Numerals adjacent to strain names represent the number of unique genes (determined by BLASTP) and the numerals adjacent to the brackets represent the size of the core genome of *A. baumannii* or within clonal lineages. The interior values are bootstrap probabilities based on 1000 replicates, and the tree was drawn using TreeGraph2 [101].

The phylogeny of *A. baumannii* D1279779, with respect to other sequenced *A. baumannii* isolates, was inferred using a MLST approach [55]. The allelic profile of this strain is 12-37-2-2-3-2-14, but it does not match any previously assigned STs; the closest allelic profile in the MLST database belonged to an ST117 isolate (12-37-2-2-9-2-14), differing only in the *recA* allele. This MLST-based analysis suggested that the nearest phylogenetic relatives of D1279779 were *A. baumannii* strains of the international clonal (IC) lineage II, though this strain did not fall within either the *A. baumannii* ICI or ICII lineages (Figure 2). This observation was congruent with our own previous PCR typing, which indicated this strain did not belong to any of the three major *A. baumannii* lineages [62]. The phylogenetic relationship of D1279779 to other *A. baumannii* strains was also consistent with the notion that community-acquired isolates are epidemiologically distinct from nosocomial isolates [26].

**Figure 3:**
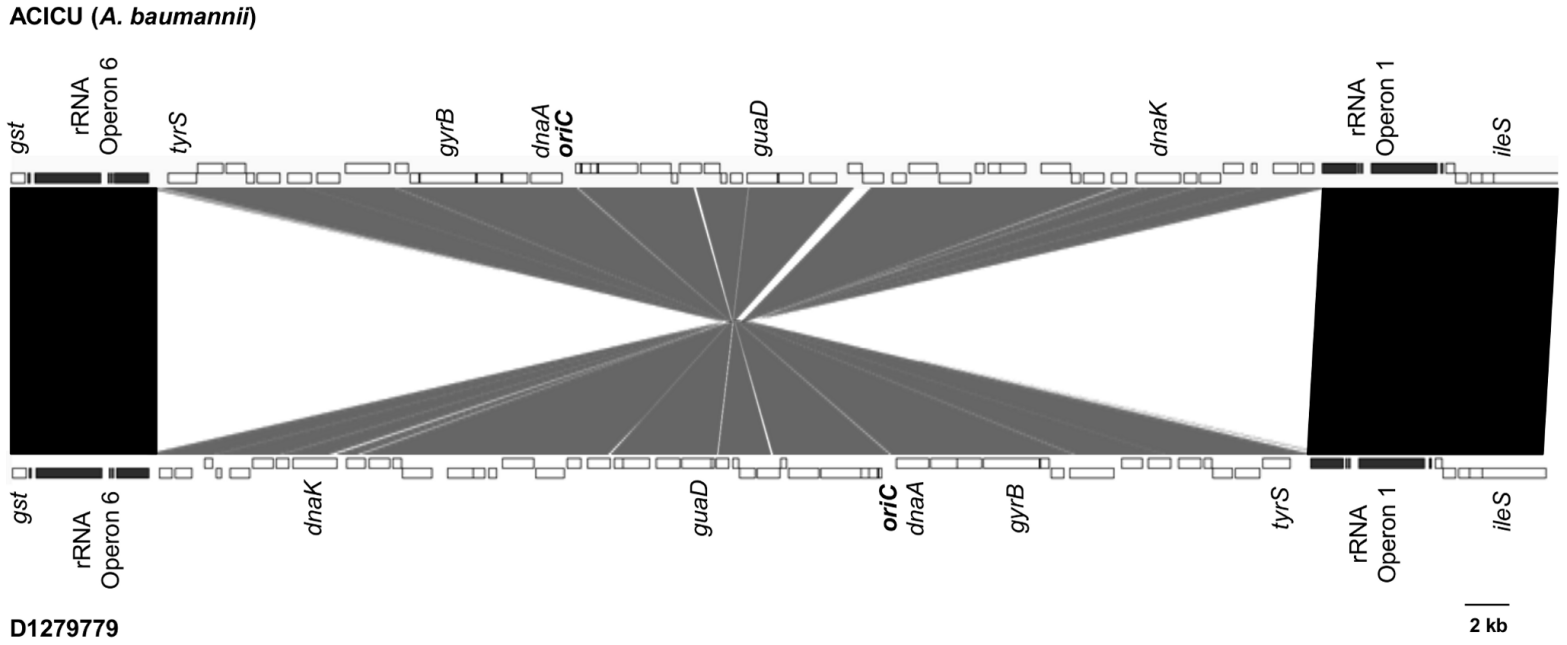
50kb inversion in the genome of *Acinetobacter baumannii* D1279779. The DNA region between rRNA operons one and six in *A. baumannii* D1279779 is conserved (≥96% nucleotide identity) but is inverted relative to other *A. baumannii* genomes (represented by ACICU), as depicted by the grey shading. Genes on positive and negative strands are depicted on the top and bottom row of rectangles, respectively. Conserved regions (≥99% nucleotide identity) in the same orientation are depicted by black shading. Locations of several conserved genes and the origin of chromosomal replication (*oriC*) are indicated. This figure was generated using the combined outputs of MAUVE [42] and the Artemis Comparison Tool [102].

Genomic alignments using MAUVE indicated that *A. baumannii* D1279779 shares a high degree of genome synteny with other completely sequenced strains of *A. baumannii*, with the exception of *A. baumannii* SDF (Figure S1), which is known to have undergone both extensive genome reduction and rearrangement [11]. In *A. baumannii* D1279779, a 50.8 kb region of sequence situated between the first and sixth rRNA operons was inverted relative to other *A. baumannii* genomes (Figure 3). We confirmed this rearrangement in D1279779 by PCR analysis conducted on the original cryogenic stock. The inversion of this region resulted in reversing the orientation of a number of critical housekeeping genes as well as the origin of chromosomal replication (*oriC*) (Figure 3). It is of note that the opposing directionality of both rRNA operons flanking the inversion was maintained, which would otherwise potentially result in replicative blockage [63]. Presumably this genomic rearrangement was mediated by homologous recombination between the two oppositely oriented rRNA operons [64]. Such rRNA operon-mediated rearrangements in genome architecture have been known to occur in other organisms and between more distal rRNA operons [64], [65], resulting in even larger inversions than observed here.

### The core and accessory genome of *A. baumannii*

The predicted proteome for all currently complete genomes of *A. baumannii* was compared by means of a reciprocal BLASTP search, which enabled estimation of the *A. baumannii* core genome size. The numbers overlaid on the phylogenetic tree in Figure 2 (adjacent to the brackets) indicate the various sizes of the core genome. A total of 1944 predicted CDS are shared between all *A. baumannii* genomes in this analysis; this increases to 2603 CDS when strain SDF is excluded from this analysis group (Figure 2). The lineage-specific core genomes of ICI and ICII is larger than the *A. baumannii* core genome, with 3098 and 3167 CDS shared respectively (Figure 2), supporting the notion that ICI and ICII constitute recent clonal lineages [55]. *A. baumannii* strains ATCC 17978 and SDF were the two deepest branching strains in the phylogenetic tree, each displaying over 500 strain-specific genes (Figure 2). *A. baumannii* D1279779 encoded 172 unique genes not present in the other sequenced strains. The majority of these genes were associated with features indicative of recent lateral acquisition, such as atypical trinucleotide composition and the presence of mobile genetic elements.

Eighteen copies of the insertion sequence IS*Aba13* and twenty-four regions of genomic plasticity (RGPs) were identified in the genome of D1279779, including two prophages and four genomic islands (Table 2). Unlike RGPs located in genomes of nosocomial isolates, those in D1279779 do not appear to encode known antibiotic resistance or virulence-associated functions (Table 2). One RGP of interest, D1279779_RGP05, contains genes encoding a type I DNA restriction-modification system and genes associated with the catabolic degradation of nitrogenous compounds. Analysis indicated the gene encoding the specificity subunit (*hsdS*) of the restriction-modification system contained a frameshift mutation. However, a truncated *hsdS* can still potentially translate and dimerize into a functional HsdS subunit, albeit with an altered DNA specificity [66]-[68]. Nevertheless, the presence of a DNA restriction-modification system in this genomic island may act as a ‘selfish’ genetic element to ensure its dissemination [69] and/or may function in bacteriophage defence and hindrance of lateral gene transfer [70].

**Table 2:** Accessory elements present in the *Acinetobacter baumannii* D1279779 genome.

(D1279779_)	CDS (ABD1_)	Size (kb)	G+C (%)	Putative function/features of interest	Integrase (target)	ATC^a^	*A. baumannii* orthologues
RGP01	00530–00630	12.1	31.3	polysaccharide biosynthesis	–	Y	ABTJ_03749–03760
RGP02	00790–00890	12.5	39.0	unknown	–	N	variable region
RGP03	01090–01140	5.9	36.8	metabolism	–	N	ABTJ_03697–03701
RGP04	04780–04960	16.6	41.2	fatty acid biosynthesis	–	N	ACICU_00517– 00535
RGP05	05270–05690	47.3	37.3	genomic island, type I restriction-modification, metabolic augmentation	Y (*dusA*)	Y	unique
RGP06	06860–07920	5.7	30.4	unknown	–	Y	unique
RGP07	08570–08650	10.0	33.8	cryptic genomic island	Y (ND^b^)	N	unique
RGP08	09940–10530	50.6	38.4	prophage, phosphoethanolamine transferase, IS*Aba13*_2	Y (ND^b^)	Y	pA_ICU_9^c^ All ex. AB0057, ATCC 17978, SDF
RGP09	10860–10930	1.7	37.5	lipoproteins	–	N	unique
RGP10	11180–11850	48.0	38.9	prophage, DNA polymerase V	Y (*lysC*)	N	A1S_1142-75 AB57_1310-1224
RGP11	12360–12570	19.3	32.9	temperature shock, metabolism	N	Y	All ex. SDF
RGP12	13160–13230	9.4	37.0	unknown, IS*Aba13*_4	–	N	unique
RGP13	13580–13650	6.2	32.9	unknown, IS*Aba13*_5	–	N	unique
RGP14	15000–15150	16.0	33.7	genomic island, fimbriae biogenesis	Y* (ND^b^)	Y	pA_ICU_16^c^ In all *A. baumannii*
RGP15	17780–17800	5.0	25.9	unknown	–	Y	unique
RGP16	17930–17970	3.0	32.1	unknown	–	N	unique
RGP17	18030–18070	3.6	40.5	degradative enzymes	–	N	unique
RGP18	18340–18480	14.9	37.1	fatty acid metabolism	–	Y	A1S_1813-22
RGP19	22050–22070	2.1	33.9	unknown	–	N	A1S_2209-11
RGP20	22680–22710	3.4	38.6	RNA modification	–	N	A1S_2271-73
RGP21	23900–24060	20.7	38.7	degradative enzymes	–	N	A1S_2397-2414
RGP22	28390–28410	2.9	30.5	polysaccharide biosynthesis	–	N	A1S_2896-3841
RGP23	31330–31680	35.0	35.5	fosfomycin resistance, IS*Aba13*_17	–	Y	variable region
RGP24	32600–32660	7.0	26.3	unknown	–	Y	A1S_3899-3901

a ATC - atypical trinucleotide composition; RGPs with χ2 values greater than 500.

b ND - Undetermined integrase target site.

c Accessory element designation in *A. baumannii* ACICU [9].

* Premature stop in integrase protein

Catabolism-associated genes in this RGP included a gene cluster encoding a glutamate dehydrogenase and a near-complete arginine succinyltransferase (AST) pathway [71]. Complete and partial copies of the AST pathway are encoded elsewhere in the core genome of *A. baumannii* D1279779. Also present in D1279779_RGP05 are genes encoding an acetylpolyamine amidohydrolase (AphA) and an allantoate amidohydrolase (AllC). AphA performs the deacetylation of varied acetylpolyamines [72], and AllC functions in the degradation of allantonate to S-ureidoglycolate [73]. The core genome of *A. baumannii* D1279779 also encodes an allantoicase (Alc) which functions in a similar manner to AllC, except degradation of allantoate is performed by a single-step catalytic mechanism, rather than a dual-step one [73].

Two other RGPs, D1279779_RGP01 and D1279779_RGP22, encode enzymes involved in capsule polysaccharide biosynthesis. The RGP D1279779_RGP03 encodes additional copies of three enzymes (diaminopimelate decarboxylase, a ribulose-phosphate 3-epimerase, and an uroporphyrinogen decarboxylase) encoded elsewhere in the core genome.

The D1279779 genome encodes two putative prophages, one of which encodes paralogues of the error-prone DNA polymerase V subunits UmuC and UmuD; these genes are frequently associated with prophages and other mobile genetic elements such as genomic islands and plasmids [74]. The second prophage encodes a paralogue of lipid A phosphoethanolamine transferase (EptA) which facilitates the covalent modification of lipid A. Overexpression of *eptA* in *A. baumannii* has previously been associated with increased colistin resistance [75]. However, strain D1279779 appears to exhibit greater colistin sensitivity than nosocomial isolates (discussed below).

Notably, *A. baumannii* D1279779 does not encode the AbaR genomic island (Table 3), a drug resistance island present in the majority of published *A. baumannii* genomes. AbaRs are characteristically found inserted within the *comM* gene [76], and associated with the accretion of multiple insertion sequences and genomic islands [77], [78]. When present, AbaRs are capable of encoding resistance to a multitude of antibiotics (aminoglycosides, beta-lactams, sulphonamides, tetracyclines) and heavy metals (arsenic and mercury) [2]. *A. baumannii* D1279779 has an intact *comM* gene and lacks an AbaR island, which may partially explain the antimicrobial susceptibility phenotype observed for this CA-AB isolate.

**Table 3:** Accessory elements absent in the *Acinetobacter baumannii* D1279779 genome.

RGP_	D1279779 boundaries	ACICU CDs	Putative function/features of interest	No. of strains with RGP present^c^
D01	02020 (*comM*)	AB57_0243-0306^a^	*A. baumannii* antibiotic resistance island	9/10 strains AbaG1 in SDF
D02	06960–06970	00685–00701	glucose dehydrogenase 2, various insertion sequences	5/10 strains exclusive to ICII
D03	07900–07910	00873–00880	haem degradation, iron acquisition	5/10 strains
D04	11060–11100	AB57_1176–1215^b^	glucarate degradation pathway, vanillate degradation	3/10 strains mediated by IS*Aba13*_3
D05	12530–12580	AB57_1379–1406^b^	degraded prophage	3/10 strains exclusive to ICI
D06	13150–13240	01295–01313	type VI secretion system	9/10 strains replaced with D1279779_RGP12
D07	13570–13660	01354–01403	β-ketoadipate pathway, 2-aminoethylphosphonate transport/metabolism, carnitine degradation	9/10 strains replaced with D1279779_RGP13
D08	17660–17960	01810–01823	pillin biogenesis, saccharopine dehydrogenase, monoamine oxidase	8/10 strains exclusive to ICI and ICII
D09	17770–17810	01833–01838	unknown	5/10 strains exclusive to ICII replaced with D1279779_RGP15
D10	17920–17980	01852–01863	unknown	5/10 strains Exclusive to ICII replaced with D1279779_RGP16
D11	18260–18270	01892–01897	demethylmenaquinone methyltransferase, phosphoglycerate dehydrogenase	5/10 strains exclusive to ICII mediated by IS*Aba13*_3
D12	18390–18490	01914–01920	metabolism	7/10 strains replaced with D1279779_RGP18
D13	18590–18610	01937–01949	various dehydrogenases	5/10 strains exclusive to ICII
D14	20390–20400	02140–02235	prophage	4/10 strains
D15	21340–21350	ABK1_1354–1403^b^	prophage	3/10 strains
D16	21980–21990	AB57_2539–2553^b^	two component regulation, fatty acid modification	3/10 strains exclusive to ICI
D17	22190–22200	AB57_2575–2579^b^	unknown	3/10 strains exclusive to ICI
D18	23890–24070	02595–02623	transcriptional regulation, transporters/permeases	7/10 strains replaced with D1279779_RGP21
D19	24530–24540	AB57_2901–2906^b^	crispr-associated proteins	3/10 strains exclusive to ICI
D20	28500–28510	03158–03160	hypothetical proteins	4/10 strains

a 3’ *comM* fragment not present in *A. baumannii* ACICU.

b Gene cluster not present in *A. baumannii* ACICU.

c Number of currently completely sequenced *A. baumannii* strains other than D1279779.

Nineteen other gene clusters, present in the genomes of other *A. baumannii* isolates, are also absent from D1279779. Ten of the missing gene clusters are unique to *A. baumannii* strains of the ICI and ICII lineages. Six other gene clusters absent in D1279779 were replaced with RGPs unique to this strain or additionally present only in ATCC 17978 (Table 2, Table 3). Some of the absent genes included those involved in the β-ketoadipate pathway [79], the D-glucarate degradation pathway [80] and potentially, the catabolism of carnitine and vanillate (Table 3). The loss of these metabolic genes very likely correlates with the observed inability of this strain to utilise glactarate, glucarate and 4-hydroxybenoazte as carbon sources but does not prevent the utilisation of carnitine (see below).

Comparison of known or predicted virulence genes amongst *A. baumannii* sequences indicated strain D1279779 lacks several genes conserved amongst previously sequenced *A. baumannii* strains. These include a type VI secretion system gene cluster [81] (Table 3), a gene encoding the *Acinetobacter* trimeric autotransporter protein (Ata) [82] (which is truncated in D1279779), haem acquisition (Table 3) and a gene encoding for the biofilm-associated protein (Bap) [83], [84]. The loss of these genes may correlate with the observation that D1279779 has only a modest capacity for biofilm formation and adherence to nasopharyngeal cells [62]. Nevertheless, this strain still carries other potential virulence-associated genes, including those coding for acinetobactin biosynthesis [85], capsular polysaccharide polymerisation/export [86], type I and type IV pili biogeneses [87] and phospholipases C and D [88], [89].

### Multiple copies of IS*Aba13* are present in the genome

Eighteen copies of the transposon IS*Aba13*, previously identified in *A. baumannii* AB0057 [8], are present throughout the D1279779 chromosome (Figure 1). All insertion sequences, with the exception of one, are of an identical isoform (Table 4). The two insertion sequence isoforms have nucleotide identities of 99% and 96% to the IS*Aba13* in AB0057. Thirteen copies of IS*Aba13* are inserted within annotated genes, including some potentially encoding virulence and competence functions such as a fimbrial adhesin, a type I secretion domain protein, bacterial capsule synthesis protein and competence-damaged induced protein CinA (Table 4). One of the copies of IS*Aba13* replaced an approximately 44 kb region, present in some other *A. baumannii* strains, that carries genes for the catabolism of glucarate, galactarate and vanillate (Table 3, Table 4).

**Table 4:** Genomic coordinates of IS*Aba13* copies and orthologous genes disrupted.

IS*Aba*13_	Coordinates	Gene(s) disrupted	Annotation/putative function	*A. baumannii* orthologues(s)
1	583114–584152	ABD1_05170	TetR family transcriptional regulator	ACICU_00556
2	1142807–1143845	ABD1_10420	prophage-associated hypothetical	ACICU_01061
3	1214764–1213726	Loss of ∼44 kb	glucarate/galactarate/vanillate catabolism	AB57_1176-1212^b^
4	1421673–1422711	ABD1_13230; 13240	hypothetical protein; ankryin repeat-containing protein	no orthologue^c^; ACICU_01314
5	1463547–1464585	ABD1_13650	hypothetical protein	no orthologue^c^
6	1487979–1489017	ABD1_13850	competence-damage inducible protein CinA	ACICU_01424
7^a^	1544569–1545607	ABD1_14440	MFS transporter	ACICU_01479
8	1787573–1788611	–	–	–
9	1892223–1891185	ABD1_17670	fimbrial family protein	ACICU_01810
10	1963590–1964628	ABD1_18260	type 1 secretion C-terminal target domain	ACICU_01891
11	2095736–2094698	–	–	–
12	2195861–2194823	–	–	–
13	2233207–2234245	–	–	–
14	2256425–2257463	–	–	–
15	2640973–2642011	ABD1_24300	hypothetical protein	ACICU_02649
16	2879635–2878597	ABD1_26440	bacterial capsule synthesis protein	ACICU_02936
17	3422518–3423556	ABD1_31460	hypothetical protein	A1S_3893^b^
18	3599602–3600640	ABD1_33030	hypothetical protein	ACICU_03597

a IS*Aba13* sequence isoform 2.

b gene(s) not present in *A. baumannii* ACICU.

c Not present in any currently complete *A. baumannii* genome.

### The catabolic phenome of *A. baumannii* D1279779

Biolog Phenotype MicroArrays are a respiration-based assay system that can test up to 2000 phenotypic traits simultaneously [56], [90]. This system uses 96 well plates with each well testing a separate phenotype using a tetrazolium dye that produces a colour change in response to cellular respiration. The phenome of *A. baumannii* D1279779 was investigated with the Biolog Phenotype MicroArray System and compared with an ICI strain (AYE), an ICII strain (ACICU) and ATCC 17978, a nosocomial isolate from 1951, predating the emergence of the major global clonal lineages as the dominant nosocomial strains.

**Figure 4:**
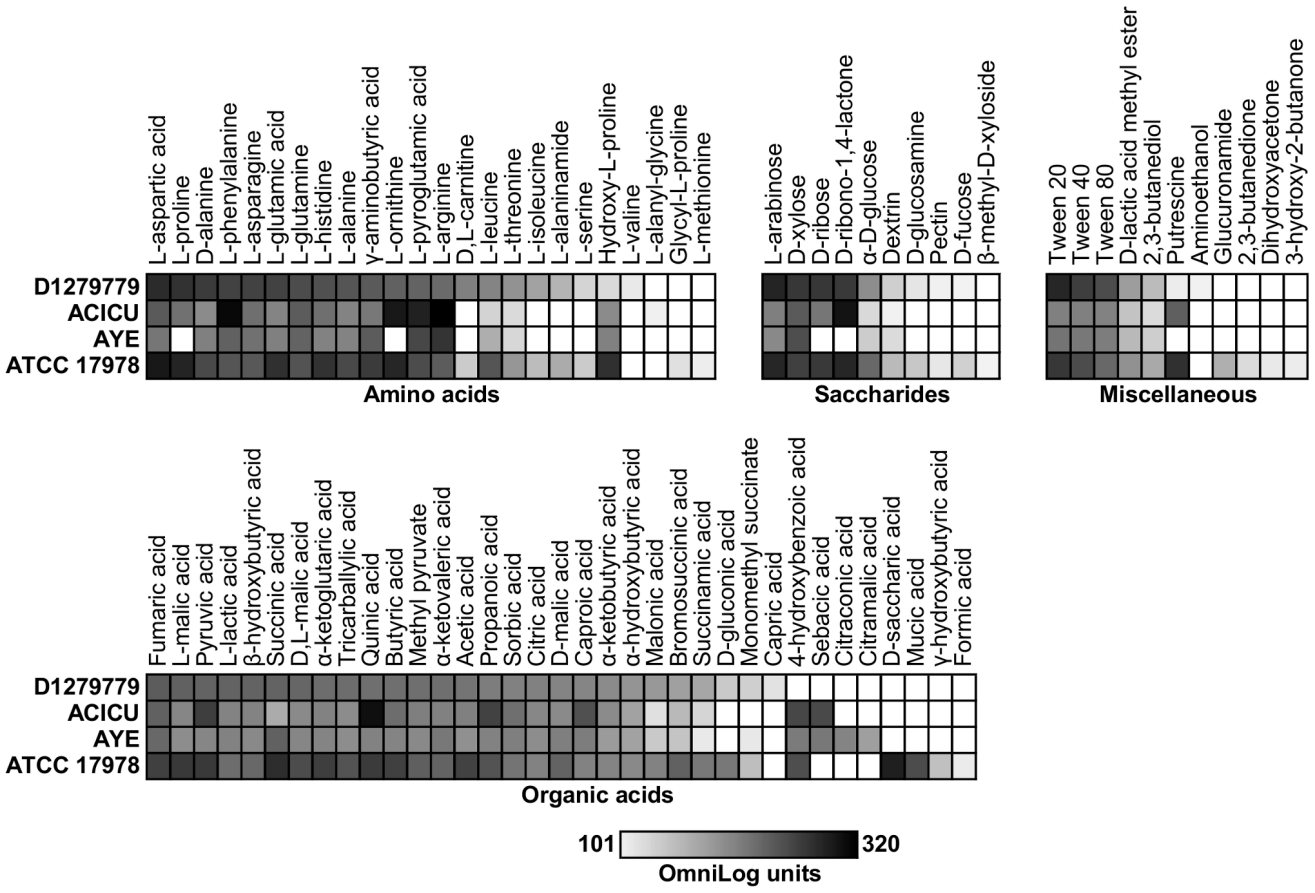
The catabolic phenome of *Acinetobacter baumannii*. Strengths of carbon utilisation phenotypes of *A. baumannii* strains D1279779, ACICU, AYE and ATCC 17978 were determined using Biolog Phenotype Microarray plates PM1 and PM2. The maximal kinetic curve height was expressed as a greyscale ranging from 101 (light grey) to 320 OmniLog units (black). Phenotypes are arranged from strongest to weakest relative to *A. baumannii* D1279779. Phenotypes <101 OmniLog units (white) were considered negative.

The four *A. baumannii* strains tested were similar in their utilization of sole carbon (Figure 4) and nitrogen sources (Figure S2). They utilized a combined total of 80 carbon sources out of the 190 tested, encompassing a range of amino acids, carboxylic acids, saccharides and miscellaneous compounds (Figure 4). Strains D1279779 and ATCC 17978 were able to utilise a greater breadth of sole carbon and nitrogen sources compared to ICI and ICII strains, particularly in relation to amino acids including alaninamide, asparagine, isoleucine, glutamate and homoserine (Figure 4, Figure S2). The observed phenotypic profiles suggest the emergence of ICI and ICII lineages in nosocomial settings has coincided with a narrowing of their substrate utilisation capabilities. Furthermore, *A. baumannii* ACICU displayed a higher respiration rate on substrates arginine, ornithine, phenylalanine, pyroglutamic acid, quinic acid and ribonolactone (Figure 4), suggesting a possible specialization in terms of carbon utilization preferences.

In order to independently confirm the Biolog respiration data, the ability of *A. baumannii* D1279779 and ACICU to grow on minimal media in the presence of fifteen sole carbon compounds was tested. Turbidity consistent with cellular replication was observed in all tested carbon sources except for D-gluconic acid and α-D-glucose (see below) (Table S1), which was concordant with the phenotype microarray data.

Metabolic reconstructions for each of the four strains were undertaken to analyse whether the phenotypic differences detected could be ascribed to the presence or absence of specific genes. *A. baumannii* D1279779 was unable to utilise 4-hydroxybenzoic acid as a sole source of carbon in either the phenotype microarray (Figure 4) or minimal media (Table S1). Genomic analysis indicated that this strain lacked the *pobA* gene encoding a 4-hydroxybenzoate 3-hydroxylase (Table 3), required for the conversion of 4-hydroxybenzoic acid to protocatechuic acid [79]. Both this strain and *A. baumannii* SDF were the only genomes examined that lacked this gene.

*A. baumannii* ATCC 17978 was the only strain in the test group able to utilise the diastereomers saccharic acid (glucarate) and mucic acid (galactarate) as sole sources of carbon (Figure 4). This was attributable to the presence of a gene cluster involved in the glucarate degradation pathway [80] conserved in some other *A. baumannii* strains, but absent in D1279779, ACICU and AYE (Table 3, Table 4).

Both strains D1279779 and ATCC 17978 showed a positive result for respiration on both D-gluconic acid and α-D-glucose. Our results were concordant with the results of a previous phenotype microarray study [91], which demonstrated the capability of various *Acinetobacter* sp. (including *A. baumannii*) to respire in the presence of both these carbon sources. This is curious, since the majority of *Acinetobacter* species including *A. baumannii* have repeatedly been reported as incapable of utilising D-glucose and D-gluconate as sole carbon sources [92], [93]. All four strains encode the Entner-Doudoroff (ED) pathway, an alternative glucose assimilation route that requires the cofactor pyrroloquinoline quinone (PQQ) [94]; a PQQ biosynthetic pathway is evident in three of the strains (but not in ATCC 17978). Growth experiments in both M9 and IF-0 minimal media using D-gluconic acid and α-D-glucose as sole carbon sources, with or without PQQ supplementation, were all negative. The apparent contradiction between the genome, phenotype microarray data and growth assays may indicate these substrates are not assimilated, but rather act as energy donors [95].

A number of the differences observed in carbon and nitrogen utilization between the strains could not be accounted for at the genetic level. This could be due to differences in regulation, membrane transport activity, or the presence of novel uncharacterized catabolic pathways. For instance, strains D1279779 and ATCC 17978 were able to utilize the branched-chain amino acids leucine and isoleucine, while ACICU and AYE were only able to respire weakly on leucine. All of the strains encoded a putative branched-chain amino acid aminotransferase (IlvE) for the reversible transamination of isoleucine, leucine and valine [96], it seems likely the phenotypic differences are due to altered regulation or transport factors. It is possible the residual leucine utilisation in AYE and ACICU is due to a tyrosine aminotransferase (TyrB), which overlaps IlvE in specificity to leucine [97]. In another instance, *A. baumannii* AYE was found to be unable to utilise proline, ornithine and putrescine as carbon sources (Figure 4) or citrulline, ornithine and putrescine as nitrogen sources (Figure S2). This suggests potential defects in proline and arginine catabolism, but these deficiencies cannot currently be accounted for at the genetic level.

### The resistance phenome of *A. baumannii* D1279779

**Figure 5:**
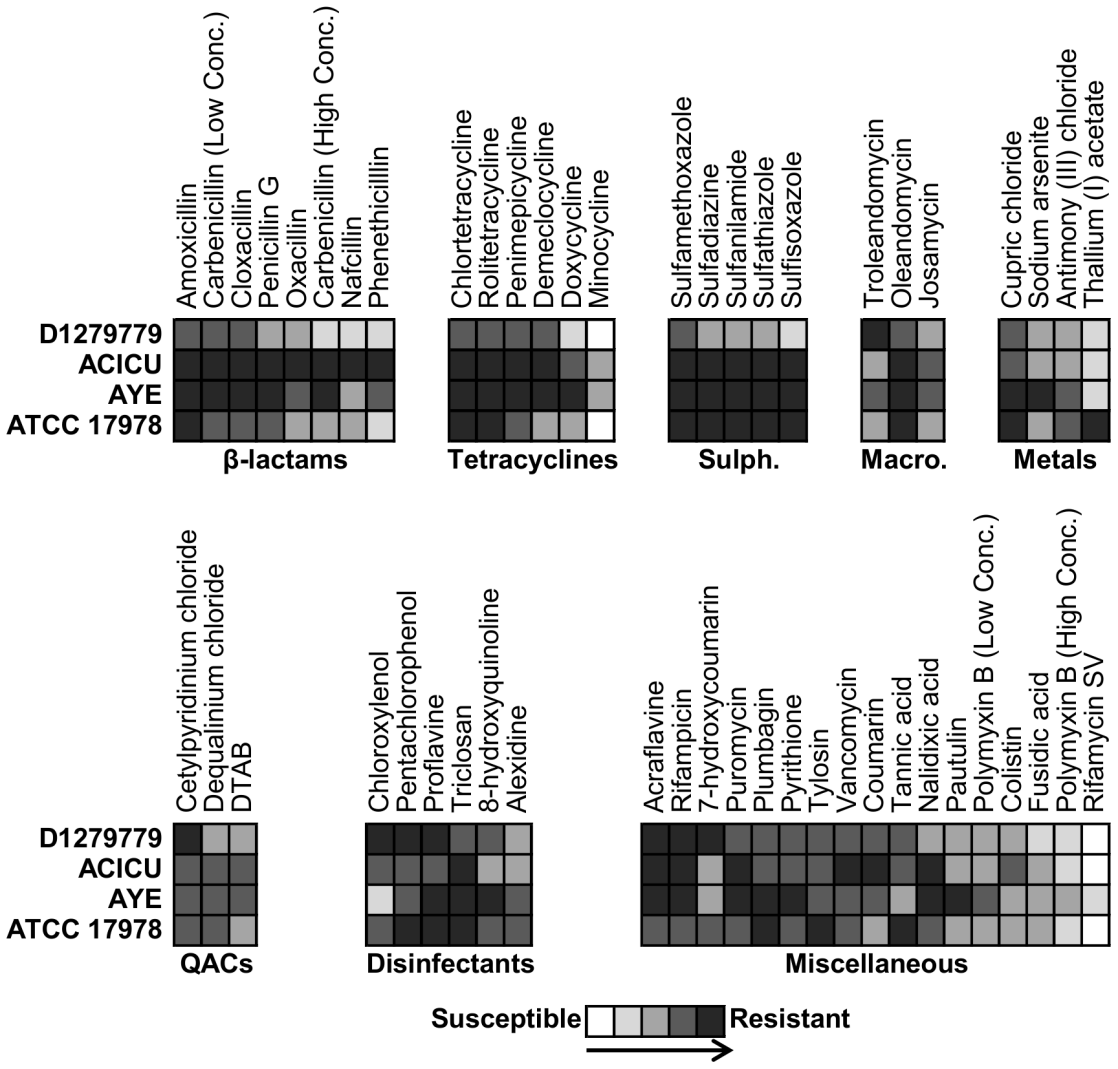
The resistance phenome of *Acinetobacter baumannii*. Select antimicrobial resistance phenotypes of *A. baumannii* strains D1279779, ACICU, AYE and ATCC 17978 are displayed as a five-coloured grey scale ranging from no resistance (white) to maximal resistance (dark grey). Phenotypes are arranged from strongest to weakest relative to *A. baumannii* D1279779. Abbreviations: Macro, macrolides; QACs, quaternary ammonium compounds; and Sulph, sulphonamides.

The osmotolerance (Table S1), pH tolerance (Figure S2) and antimicrobial resistance (Figure 5, Table S1) of the four *A. baumannii* strains was also examined with Biolog Phenotype MicroArrays. *A. baumannii* ACICU and ATCC 17978 were found to be more sensitive to acidic pH and were only able to deaminate a limited number of compounds at pH 4.5 (Figure S2).

The four *A. baumannii* strains displayed high intrinsic resistance to many antimicrobial compounds. Respiration in all four strains was observed, at all concentrations, for 94 of the 240 antimicrobials tested (Table S1). No respiration was observed in any strain for five compounds: the antibiotic novobiocin and the heavy metals salts potassium chromate, cadmium chloride, sodium orthovanadate and sodium metavanadate. Differential susceptibility to a further 106 compounds was observed in the four strains (Figure 5, Table S1). Strains D1279779 and ATCC 17978 were noticeably more susceptible to a range of clinically important antibiotics, including the beta-lactams and tetracyclines. The higher levels of resistance of *A. baumannii* ACICU and AYE towards beta-lactams and sulphonamides is likely due to resistance determinants encoded within their respective AbaR elements [2], [9], as well as the carbapenem resistance plasmid pACICU1 [9]. Although, ATCC 17978 was also resistant to sulphonamides, it is likely that this resistance is encoded on a separate genomic island [98]. The strain AYE encodes two TetA tetracycline resistance efflux pumps [2], though in the case of ACICU, there are no characterized tetracycline resistance genes; the observed resistance may be due to the function of other efflux pumps. Resistance to the quinolone nalidixic acid can be accounted for by mutations both in *gyrA* and *parC* in strain AYE, and a mutation in *gyrA* for strain ACICU [8]; neither mutation is present in ATCC 17978 or D1279779. *A. baumannii* AYE was found to be relatively more resistant to arsenic and rifamycin SV, attributable to the respective presence of an arsenic resistance cluster and a rifampin ADP-ribosyltransferase (*arr-2*) in its AbaR island [2]. Strains AYE and ATCC 17978 had increased resistance to copper, very likely due to the presence of a copper resistance cluster (*copABCDRS*) [8], in addition to the copper resistance genes (*pcoAB*) present in the *A. baumannii* core genome.

## Conclusions

The CA-AB isolate D1279779, while phylogenetically related to the ICII *A. baumannii* global clonal lineage, phenotypically resembles ATCC 17978 in terms of carbon and nitrogen utilization, and drug susceptibility profile. Phenotypic testing of nosocomial *A. baumannii* suggests that the narrowing of substrate utilisation capabilities and expansion of drug resistance profiles in both ICI and ICII global clonal lineages has contributed to their success in the nosocomial milieu. Our genomic analysis of the CA-AB isolate D1279779 reveals the absence of the AbaR island common to nosocomial isolates. D1279779 does however comprise of 24 novel RGPs that encode catabolic functions, polysaccharide biosynthesis and many hypothetical proteins of unknown function. Reports in the literature have suggested that CA-AB is associated with higher mortality rates than nosocomial *A. baumannii* strains. Whilst there were no obvious virulence-associated genes unique to D1279779, there was however, the apparent loss of several genes associated with virulence, particularly with respect to biofilm formation and eukaryotic cell adhesion. The characteristics of D1279779 may be more representative of an environmental or pre-antibiotic era clinical *A. baumannii* isolate, and appears quite distinct from the current dominant lineages of nosocomial isolates.

## Supporting Information

Figure S1 **Synteny of** ***Acinetobacter baumannii*** **.** Chromosomal alignments of the *A. baumannii* D1279779 genome against the ten currently complete *A. baumannii* genomes were generated using progressive MAUVE [103]. Regions of significant synteny between the strains are shown as coloured blocks and unshared regions are seen as white gaps.
(PNG)

Figure S2 **Phenotypic analysis of nitrogen utilisation and pH stress tolerance.** Strengths of nitrogen utilisation (A) and the pH tolerance phenotypes (B) of *A. baumannii* strains D1279779, ACICU, AYE and ATCC 17978 were determined were determined using Biolog Phenotype Microarray plates PM3 and PM10, respectively. The maximal kinetic curve height was expressed as a greyscale ranging from 101 OmniLog units (light grey) to 310 and 360 OmniLog units (black) for nitrogen and pH tolerance phenotypes, respectively. Phenotypes are arranged from strongest to weakest relative to *A. baumannii* D1279779. Phenotypes <115 OmniLog units for nitrogen phenotypes and <101 OmniLog units for pH tolerance phenotypes were considered negative phenotypes and are represented in white.
(TIF)

Table S1 **Raw phenotype microarray data.** The maximal kinetic curve height for all phenotypes obtained from plates PM1-3 (carbon and nitrogen utilisation) and PM9-20 (osmotolerance, pH tolerance and antimicrobial exposure) expressed in OmniLog units.
(XLS)

## References

[1] Roca I, Espinal P, Vila-Farres X, Vila J (2012) The *Acinetobacter baumannii* oxymoron: commensal hospital dweller turned pan-drug-resistant menace. Front Microbiol 3: 148. 22536199 10.3389/fmicb.2012.00148 PMC3333477

[2] Fournier PE, Vallenet D, Barbe V, Audic S, Ogata H, et al (2006) Comparative genomics of multidrug resistance in *Acinetobacter baumannii* . PLoS Genet 2: 62–72. 10.1371/journal.pgen.0020007 PMC1326220 16415984

[3] Vila J, Marti S, Sanchez-Cespedes J (2007) Porins, efflux pumps and multidrug resistance in *Acinetobacter baumannii* . J Antimicrob Chemoth 59: 1210–1215. 10.1093/jac/dkl509 17324960

[4] Wendt C, Dietze B, Dietz E, Ruden H (1997) Survival of *Acinetobacter baumannii* on dry surfaces. J Clin Microbiol 35: 1394–1397. 9163451 10.1128/jcm.35.6.1394-1397.1997 PMC229756

[5] Jawad A, Seifert H, Snelling AM, Heritage J, Hawkey PM (1998) Survival of *Acinetobacter baumannii* on dry surfaces: comparison of outbreak and sporadic isolates. J Clin Microbiol 36: 1938–1941. 9650940 10.1128/jcm.36.7.1938-1941.1998 PMC104956

[6] Neely AN, Maley MP, Warden GD (1999) Computer keyboards as reservoirs for *Acinetobacter baumannii* in a burn hospital. Clin Infect Dis 29: 1358–1359. 10.1086/313463 10525257

[7] Park JY, Kim S, Kim S-M, Cha SH, Lim S-K, et al (2011) Complete genome sequence of multidrug-resistant *Acinetobacter baumannii* strain 1656-2, which forms sturdy biofilm. J Bacteriol 193: 6393–6394. 22038960 10.1128/JB.06109-11 PMC3209198

[8] Adams MD, Goglin K, Molyneaux N, Hujer KM, Lavender H, et al (2008) Comparative genome sequence analysis of multidrug-resistant *Acinetobacter baumannii* . J Bacteriol 190: 8053–8064. 18931120 10.1128/JB.00834-08 PMC2593238

[9] Iacono M, Villa L, Fortini D, Bordoni R, Imperi F, et al (2008) Whole-genome pyrosequencing of an epidemic multidrug-resistant *Acinetobacter baumannii* strain belonging to the European clone II group. Antimicrob Agents Chemother 52: 2616–2625. 18411315 10.1128/AAC.01643-07 PMC2443898

[10] Smith MG, Gianoulis TA, Pukatzki S, Mekalanos JJ, Ornston LN, et al (2007) New insights into *Acinetobacter baumannii* pathogenesis revealed by high-density pyrosequencing and transposon mutagenesis. Gene Dev 21: 601–614. 17344419 10.1101/gad.1510307 PMC1820901

[11] Vallenet D, Nordmann P, Barbe V, Poirel L, Mangenot S, et al (2008) Comparative analysis of *Acinetobacters*: three genomes for three lifestyles. PLoS One 3: e1805. 18350144 10.1371/journal.pone.0001805 PMC2265553

[12] Gao F, Wang Y, Liu Y-J, Wu X-M, Lv X, et al (2011) Genome sequence of *Acinetobacter baumannii* MDR-TJ. J Bacteriol 193: 2365–2366. 21398552 10.1128/JB.00226-11 PMC3133082

[13] Zhou H, Zhang T, Yu D, Pi B, Yang Q, et al (2011) Genomic analysis of the multidrug-resistant *Acinetobacter baumannii* strain MDR-ZJ06 widely spread in China. Antimicrob Agents Chemother 55: 4506–4512. 21788470 10.1128/AAC.01134-10 PMC3187012

[14] Chen C-C, Lin Y-C, Sheng W-H, Chen Y-C, Chang S-C, et al (2011) Genome sequence of a dominant, multidrug-resistant *Acinetobacter baumannii* strain, TCDC-AB0715. J Bacteriol 193: 2361–2362. 21398540 10.1128/JB.00244-11 PMC3133099

[15] Adams MD, Chan ER, Molyneaux ND, Bonomo RA (2010) Genomewide analysis of divergence of antibiotic resistance determinants in closely related isolates of *Acinetobacter baumannii* . Antimicrob Agents Chemother 54: 3569–3577. 20530228 10.1128/AAC.00057-10 PMC2934971

[16] La Scola B, Raoult D (2004) *Acinetobacter baumannii* in human body louse. Emerg Infect Dis 10: 1671–1673. 15498175 10.3201/eid1009.040242 PMC3320269

[17] Bouvresse S, Socolovshi C, Berdjane Z, Durand R, Izri A, et al (2011) No evidence of *Bartonella quintana* but detection of *Acinetobacter baumannii* in head lice from elementary schoolchildren in Paris. Comp Immunol Microb 34: 475–477. 10.1016/j.cimid.2011.08.007 21974965

[18] Sarma PM, Bhattacharya D, Krishnan S, Lal B (2004) Assessment of intra-species diversity among strains of *Acinetobacter baumannii* isolated from sites contaminated with petroleum hydrocarbons. Can J Microbiol 50: 405–414. 15284886 10.1139/w04-018

[19] Chang LK, Ibrahim D, Omar IC (2011) A laboratory scale bioremediation of Tapis crude oil contaminated soil by bioaugmentation of *Acinetobacter baumannii* T30C. Afr J Microbiol Res 5: 2609–2615.

[20] Berg G, Roskot N, Steidle A, Eberl L, Zock A, et al (2002) Plant-dependent genotypic and phenotypic diversity of antagonistic rhizobacteria isolated from different *Verticillium* host plants. Appl Environ Microb 68: 3328–3338. 10.1128/AEM.68.7.3328-3338.2002 PMC126805 12089011

[21] Sachdev D, Nema P, Dhakephalkar P, Zinjarde S, Chopade B (2010) Assessment of 16S rRNA gene-based phylogenetic diversity and promising plant growth-promoting traits of *Acinetobacter* community from the rhizosphere of wheat. Microbiol Res 165: 627–638. 20116982 10.1016/j.micres.2009.12.002

[22] Girlich D, Poirel L, Nordmann P (2010) First isolation of the blaOXA-23 carbapenemase gene from an environmental *Acinetobacter baumannii* isolate. Antimicrob Agents Chemother 54: 578–579. 19884362 10.1128/AAC.00861-09 PMC2798507

[23] Ghai R, Rodriguez-Valera F, McMahon KD, Toyama D, Rinke R, et al (2011) Metagenomics of the water column in the pristine upper course of the Amazon River. PLoS One 6: e23785. 21915244 10.1371/journal.pone.0023785 PMC3158796

[24] Chu YW, Leung CM, Houang ET, Ng KC, Leung CB, et al (1999) Skin carriage of Acinetobacters in Hong Kong. J Clin Microbiol 37: 2962–2967. 10449482 10.1128/jcm.37.9.2962-2967.1999 PMC85423

[25] Anstey NM, Currie BJ, Hassell M, Palmer D, Dwyer B, et al (2002) Community-acquired bacteremic *Acinetobacter* pneumonia in tropical Australia is caused by diverse strains of *Acinetobacter baumannii*, with carriage in the throat in at-risk groups. J Clin Microbiol 40: 685–686. 11825997 10.1128/JCM.40.2.685-686.2002 PMC153418

[26] Zeana C, Larson E, Sahni J, Bayuga SJ, Wu F, et al (2003) The epidemiology of multidrug-resistant *Acinetobacter baumannii*: Does the community represent a reservoir? Infect Cont Hosp Ep 24: 275–279. 10.1086/502209 12725357

[27] Chen HP, Chen TL, Lai CH, Fung CP, Wong WW, et al (2005) Predictors of mortality in *Acinetobacter baumannii* bacteremia. J Microbiol Immunol Infect 38: 127–136. 15843858

[28] Falagas ME, Karveli EA, Kelesidis I, Kelesidis T (2007) Community-acquired *Acinetobacter* infections. Eur J Clin Microbiol 26: 857–868. 10.1007/s10096-007-0365-6 17701432

[29] Elliott JH, Anstey NM, Jacups SP, Fisher DA, Currie BJ (2005) Community-acquired pneumonia in northern Australia: low mortality in a tropical region using locally-developed treatment guidelines. Int J Infect Dis 9: 15–20. 15603991 10.1016/j.ijid.2004.09.008

[30] Leung AS, Chu CM, Tsang KY, Lo FH, Lo KF, et al (2006) Fulminant community-acquired *Acinetobacter baumannii* pneumonia as a distinct clinical syndrome. Chest 129: 102–109. 16424419 10.1378/chest.129.1.102

[31] Wang JT, McDonald LC, Chang SC, Ho M (2002) Community-acquired *Acinetobacter baumannii* bacteremia in adult patients in Taiwan. J Clin Microbiol 40: 1526–1529. 11923388 10.1128/JCM.40.4.1526-1529.2002 PMC140383

[32] Ong CWM, Lye DCB, Khoo KL, Chua GSW, Yeoh SF, et al (2009) Severe community–acquired *Acinetobacter baumannii* pneumonia: An emerging highly lethal infectious disease in the Asia-Pacific. Respirology 14: 1200–1205. 19909464 10.1111/j.1440-1843.2009.01630.x

[33] Chong YP, Jung K-S, Lee KH, Kim M-N, Moon SM, et al (2010) The bacterial etiology of community-acquired pneumonia in Korea: a nationwide prospective multicenter study. Infect Chemother 42: 397–403.

[34] Anstey NM, Currie BJ, Withnall KM (1992) Community-acquired *Acinetobacter* pneumonia in the Northern Territory of Australia. Clin Infect Dis 14: 83–91. 1571467 10.1093/clinids/14.1.83

[35] Gradon JD, Chapnick EK, Lutwick LI (1992) Infective endocarditis of a native valve due to *Acinetobacter*: case report and review. Clin Infect Dis 14: 1145–1148. 1600019 10.1093/clinids/14.5.1145

[36] Bick JA, Semel JD (1993) Fulminant community-acquired *Acinetobacter* pneumonia in a healthy woman. Clin Infect Dis 17: 820–821. 8268377 10.1093/clinids/17.4.820

[37] Reindersma P, Nohlmans L, Korten JJ (1993) *Acinetobacter*, an infrequent cause of community acquired bacterial meningitis. Clin Neurol Neurosurg 95: 71–73. 8453820 10.1016/0303-8467(93)90096-y

[38] Ozaki T, Nishimura N, Arakawa Y, Suzuki M, Narita A, et al (2009) Community-acquired *Acinetobacter baumannii* meningitis in a previously healthy 14-month-old boy. J Infect Chemother 15: 322–324. 19856071 10.1007/s10156-009-0704-x

[39] Moreira Silva G, Morais L, Marques L, Senra V (2012) *Acinetobacter* community-acquired pneumonia in a healthy child. Rev Port Pneumol 18: 96–98. 21963110 10.1016/j.rppneu.2011.07.006

[40] Einsiedel LJ, Woodman RJ (2010) Two nations: racial disparities in bloodstream infections recorded at Alice Springs Hospital, central Australia, 2001–2005. Med J Aust 192: 567–571. 20477732 10.5694/j.1326-5377.2010.tb03638.x

[41] Chevreux B, Wetter T, Suhai S (1999) Genome sequence assembly using trace signals and additional sequence information. Computer Science and Biology: Proceedings of the German Conference on Bioinformatics (GCB) 99: 45–56.

[42] Darling ACE, Mau B, Blattner FR, Perna NT (2004) Mauve: multiple alignment of conserved genomic sequence with rearrangements. Genome Res 14: 1394–1403. 15231754 10.1101/gr.2289704 PMC442156

[43] van Hijum SAFT, Zomer AL, Kuipers OP, Kok J (2005) Projector 2: contig mapping for efficient gap-closure of prokaryotic genome sequence assemblies. Nucleic Acids Res 33: W560–W566. 15980536 10.1093/nar/gki356 PMC1160117

[44] Altschul SF, Madden TL, Schaffer AA, Zhang JH, Zhang Z, et al (1997) Gapped BLAST and PSI-BLAST: a new generation of protein database search programs. Nucleic Acids Res 25: 3389–3402. 9254694 10.1093/nar/25.17.3389 PMC146917

[45] Hall TA (1999) BioEdit: a user-friendly biological sequence alignment editor and analysis program for Windows 95/98/NT. Nucl Acids Symp Ser 41: 95–98.

[46] Aziz RK, Bartels D, Best AA, DeJongh M, Disz T, et al (2008) The RAST server: Rapid Annotations using Subsystems Technology. BMC Genomics 9: 75. 18261238 10.1186/1471-2164-9-75 PMC2265698

[47] Rutherford K, Parkhill J, Crook J, Horsnell T, Rice P, et al (2000) Artemis: sequence visualization and annotation. Bioinformatics 16: 944–945. 11120685 10.1093/bioinformatics/16.10.944

[48] Okonechnikov K, Golosova O, Fursov M (2012) Unipro UGENE: a unified bioinformatics toolkit. Bioinformatics 28: 1166–1167. 22368248 10.1093/bioinformatics/bts091

[49] Camacho C, Coulouris G, Avagyan V, Ma N, Papadopoulos J, et al (2009) BLAST+: architecture and applications. BMC Bioinformatics 10: 421. 20003500 10.1186/1471-2105-10-421 PMC2803857

[50] Paulsen IT, Seshadri R, Nelson KE, Eisen JA, Heidelberg JF, et al (2002) The *Brucella suis* genome reveals fundamental similarities between animal and plant pathogens and symbionts. Proc Natl Acad Sci USA 99: 13148–13153. 12271122 10.1073/pnas.192319099 PMC130601

[51] Mathee K, Narasimhan G, Valdes C, Qiu X, Matewish JM, et al (2008) Dynamics of *Pseudomonas aeruginosa* genome evolution. Proc Natl Acad Sci USA 105: 3100–3105. 18287045 10.1073/pnas.0711982105 PMC2268591

[52] Ogier J-C, Calteau A, Forst S, Goodrich-Blair H, Roche D, et al (2010) Units of plasticity in bacterial genomes: new insight from the comparative genomics of two bacteria interacting with invertebrates, *Photorhabdus* and *Xenorhabdus* . BMC Genomics 11: 568. 20950463 10.1186/1471-2164-11-568 PMC3091717

[53] Siguier P, Perochon J, Lestrade L, Mahillon J, Chandler M (2006) ISfinder: the reference centre for bacterial insertion sequences. Nucleic Acids Res 34: D32–D36. 16381877 10.1093/nar/gkj014 PMC1347377

[54] Tamura K, Peterson D, Peterson N, Stecher G, Nei M, et al (2011) MEGA5: Molecular Evolutionary Genetics Analysis using maximum likelihood, evolutionary distance, and maximum parsimony methods. Mol Biol Evol 28: 2731–2739. 21546353 10.1093/molbev/msr121 PMC3203626

[55] Diancourt L, Passet V, Nemec A, Dijkshoorn L, Brisse S (2010) The population structure of *Acinetobacter baumannii*: expanding multiresistant clones from an ancestral susceptible genetic pool. PLoS One 5: e10034. 20383326 10.1371/journal.pone.0010034 PMC2850921

[56] Bochner BR, Gadzinski P, Panomitros E (2001) Phenotype microarrays for high-throughput phenotypic testing and assay of gene function. Genome Res 11: 1246–1255. 11435407 10.1101/gr.186501 PMC311101

[57] Keseler IM, Collado-Vides J, Santos-Zavaleta A, Peralta-Gil M, Gama-Castro S, et al (2011) EcoCyc: a comprehensive database of *Escherichia coli* biology. Nucleic Acids Res 39: D583–D590. 21097882 10.1093/nar/gkq1143 PMC3013716

[58] Caspi R, Altman T, Dreher K, Fulcher CA, Subhraveti P, et al (2012) The MetaCyc database of metabolic pathways and enzymes and the BioCyc collection of pathway/genome databases. Nucleic Acids Res 40: D742–D753. 22102576 10.1093/nar/gkr1014 PMC3245006

[59] Kanehisa M, Goto S, Kawashima S, Okuno Y, Hattori M (2004) The KEGG resource for deciphering the genome. Nucleic Acids Res 32: D277–D280. 14681412 10.1093/nar/gkh063 PMC308797

[60] Bertini A, Poirel L, Mugnier PD, Villa L, Nordmann P, et al (2010) Characterization and PCR-based replicon typing of resistance plasmids in *Acinetobacter baumannii* . Antimicrob Agents Chemother 54: 4168–4177. 20660691 10.1128/AAC.00542-10 PMC2944597

[61] Dorsey CW, Tomaras AP, Actis LA (2006) Sequence and organization of pMAC, an *Acinetobacter baumannii* plasmid harboring genes involved in organic peroxide resistance. Plasmid 56: 112–123. 16530832 10.1016/j.plasmid.2006.01.004

[62] Eijkelkamp BA, Stroeher UH, Hassan KA, Papadimitrious MS, Paulsen IT, et al (2011) Adherence and motility characteristics of clinical *Acinetobacter baumannii* isolates. FEMS Microbiol Lett 323: 44–51. 22092679 10.1111/j.1574-6968.2011.02362.x

[63] Srivatsan A, Tehranchi A, MacAlpine DM, Wang JD (2010) Co-orientation of replication and transcription preserves genome integrity. PLoS Genetics 6: e1000810. 20090829 10.1371/journal.pgen.1000810 PMC2797598

[64] Hill CW, Harnish BW (1981) Inversions between ribosomal RNA genes of *Escherichia coli* . Proc Natl Acad Sci USA 78: 7069–7072. 6273909 10.1073/pnas.78.11.7069 PMC349196

[65] Klockgether J, Munder A, Neugebauer J, Davenport CF, Stanke F, et al (2010) Genome diversity of *Pseudomonas aeruginosa* PAO1 laboratory strains. J Bacteriol 192: 1113–1121. 20023018 10.1128/JB.01515-09 PMC2812968

[66] Abadjieva A, Patel J, Webb M, Zinkevich V, Firman K (1993) A Deletion Mutant of the Type-IC Restriction-Endonuclease *Eco*R124I Expressing a Novel DNA Specificity. Nucleic Acids Res 21: 4435–4443. 8233776 10.1093/nar/21.19.4435 PMC311173

[67] Meister J, MacWilliams M, Hubner P, Jutte H, Skrzypek E, et al (1993) Macroevolution by transposition: drastic modification of DNA recognition by a type I restriction enzyme following Tn5 transposition. EMBO J 12: 4585–4591. 8223468 10.1002/j.1460-2075.1993.tb06147.x PMC413889

[68] Adamczyk-Poplawska M, Lower M, Piekarowicz A (2011) Deletion of one nucleotide within the homonucleotide tract present in the *hsdS* gene alters the DNA sequence specificity of type I restriction-modification system NgoAV. J Bacteriol 193: 6750–6759. 21984785 10.1128/JB.05672-11 PMC3232900

[69] Kobayashi I (2004) Restriction-modification systems as minimal forms of life. In: Pingoud AM, editor. Restriction Endonucleases: Springer Berlin Heidelberg. pp. 19–62.

[70] Waldron DE, Lindsay JA (2006) Sau1: a novel lineage-specific type I restriction-modification system that blocks horizontal gene transfer into *Staphylococcus aureus* and between *S. aureus* isolates of different lineages. J Bacteriol 188: 5578–5585. 16855248 10.1128/JB.00418-06 PMC1540015

[71] Schneider BL, Kiupakis AK, Reitzer LJ (1998) Arginine catabolism and the arginine succinyltransferase pathway in *Escherichia coli* . J Bacteriol 180: 4278–4286. 9696779 10.1128/jb.180.16.4278-4286.1998 PMC107427

[72] Sakurada K, Ohta T, Fujishiro K, Hasegawa M, Aisaka K (1996) Acetylpolyamine amidohydrolase from *Mycoplana ramosa*: gene cloning and characterization of the metal-substituted enzyme. J Bacteriol 178: 5781–5786. 8824626 10.1128/jb.178.19.5781-5786.1996 PMC178420

[73] Agarwal R, Burley SK, Swaminathan S (2007) Structural analysis of a ternary complex of allantoate amidohydrolase from *Escherichia coli* reveals its mechanics. J Mol Biol 368: 450–463. 17362992 10.1016/j.jmb.2007.02.028

[74] Permina EA, Mironov AA, Gelfand MS (2002) Damage-repair error-prone polymerases of eubacteria: association with mobile genome elements. Gene 293: 133–140. 12137951 10.1016/s0378-1119(02)00701-1

[75] Beceiro A, Llobet E, Aranda J, Bengoechea JA, Doumith M, et al (2011) Phosphoethanolamine modification of lipid A in colistin-resistant variants of *Acinetobacter baumannii* mediated by the *pmrAB* two-component regulatory system. Antimicrob Agents Chemother 55: 3370–3379. 21576434 10.1128/AAC.00079-11 PMC3122444

[76] Bonnin RA, Poirel L, Nordmann P (2012) AbaR-type transposon structures in *Acinetobacter baumannii* . J Antimicrob Chemoth 67: 234–236. 10.1093/jac/dkr413 21965430

[77] Post V, White PA, Hall RM (2010) Evolution of AbaR-type genomic resistance islands in multiply antibiotic-resistant *Acinetobacter baumannii* . J Antimicrob Chemoth 65: 1162–1170. 10.1093/jac/dkq095 20375036

[78] Krizova L, Dijkshoorn L, Nemec A (2011) Diversity and evolution of AbaR genomic resistance islands in *Acinetobacter baumannii* strains of European clone I. Antimicrob Agents Chemother. 55: 3201–3206. 10.1128/AAC.00221-11 PMC3122396 21537009

[79] Park SH, Kim JW, Yun SH, Leem SH, Kahng HY, et al (2006) Characterization of β-ketoadipate pathway from multi-drug resistance bacterium, *Acinetobacter baumannii* DU202 by proteomic approach. J Microbiol 44: 632–640. 17205041

[80] Aghaie A, Lechaplais C, Sirven P, Tricot S, Besnard-Gonnet M, et al (2008) New insights into the alternative D-glucarate degradation pathway. J Biol Chem 283: 15638–15646. 18364348 10.1074/jbc.M800487200 PMC3259651

[81] Henry R, Vithanage N, Harrison P, Seemann T, Coutts S, et al (2012) Colistin-resistant, lipopolysaccharide-deficient *Acinetobacter baumannii* responds to lipopolysaccharide loss through increased expression of genes involved in the synthesis and transport of lipoproteins, phospholipids, and poly-β-1,6-N-acetylglucosamine. Antimicrob Agents Chemother 56: 59–69. 22024825 10.1128/AAC.05191-11 PMC3256090

[82] Bentancor LV, Camacho-Peiro A, Bozkurt-Guzel C, Pier GB, Maira-Litrán T (2012) Identification of Ata, a multifunctional trimeric autotransporter of *Acinetobacter baumannii* . J Bacteriol 194: 3950–3960. 22609912 10.1128/JB.06769-11 PMC3416510

[83] Loehfelm TW, Luke NR, Campagnari AA (2008) Identification and characterization of an *Acinetobacter baumannii* biofilm-associated protein. J Bacteriol 190: 1036–1044. 18024522 10.1128/JB.01416-07 PMC2223572

[84] Brossard KA, Campagnari AA (2012) The *Acinetobacter baumannii* biofilm-associated protein plays a role in adherence to human epithelial cells. Infect Immun 80: 228–233. 22083703 10.1128/IAI.05913-11 PMC3255684

[85] Gaddy JA, Arivett BA, McConnell MJ, López-Rojas R, Pachón J, et al (2012) Role of acinetobactin-mediated iron acquisition functions in the interaction of *Acinetobacter baumannii* strain ATCC 19606T with human lung epithelial cells, *Galleria mellonella* caterpillars, and mice. Infect Immun 80: 1015–1024. 22232188 10.1128/IAI.06279-11 PMC3294665

[86] Russo TA, Luke NR, Beanan JM, Olson R, Sauberan SL, et al (2010) The K1 capsular polysaccharide of A*cinetobacter baumannii* strain 307-0294 is a major virulence factor. Infect Immun 78: 3993–4000. 20643860 10.1128/IAI.00366-10 PMC2937447

[87] Eijkelkamp B, Hassan K, Paulsen I, Brown M (2011) Investigation of the human pathogen *Acinetobacter baumannii* under iron limiting conditions. BMC Genomics 12: 126. 21342532 10.1186/1471-2164-12-126 PMC3055841

[88] Camarena L, Bruno V, Euskirchen G, Poggio S, Snyder M (2010) Molecular mechanisms of ethanol-induced pathogenesis revealed by RNA-sequencing. PLoS Pathog 6: e1000834. 20368969 10.1371/journal.ppat.1000834 PMC2848557

[89] Jacobs AC, Hood I, Boyd KL, Olson PD, Morrison JM, et al (2010) Inactivation of phospholipase D diminishes *Acinetobacter baumannii* pathogenesis. Infect Immun 78: 1952–1962. 20194595 10.1128/IAI.00889-09 PMC2863507

[90] Johnson DA, Tetu SG, Phillippy K, Chen J, Ren Q, et al (2008) High-throughput phenotypic characterization of *Pseudomonas aeruginosa* membrane transport genes. PLoS Genet 4: e1000211. 18833300 10.1371/journal.pgen.1000211 PMC2542419

[91] Bernards AT, Dijkshoorn L, Vandertoorn J, Bochner BR, Vanboven CPA (1995) Phenotypic characterization of *Acinetobacter* strains of 13 DNA-DNA hybridization groups by means of the Biolog system. J Med Microbiol 42: 113–119. 7869346 10.1099/00222615-42-2-113

[92] Baumann P, Doudoroff M, Stanier RY (1968) A study of the Moraxella group. II. oxidative-negative species (genus *Acinetobacter*). J Bacteriol 95: 1520–1541. 5650064 10.1128/jb.95.5.1520-1541.1968 PMC252171

[93] Nemec A, Krizova L, Maixnerova M, van der Reijden TJK, Deschaght P, et al (2011) Genotypic and phenotypic characterization of the *Acinetobacter calcoaceticus*-*Acinetobacter baumannii* complex with the proposal of *Acinetobacter pittii* sp nov (formerly *Acinetobacter* genomic species 3) and *Acinetobacter nosocomialis* sp nov (formerly *Acinetobacter* genomic species 13TU). Res Microbiol 162: 393–404. 21320596 10.1016/j.resmic.2011.02.006

[94] Peekhaus N, Conway T (1998) What's for dinner?: Entner-Doudoroff metabolism in *Escherichia coli* . J Bacteriol 180: 3495–3502. 9657988 10.1128/jb.180.14.3495-3502.1998 PMC107313

[95] Müller RH, Babel W (1986) Glucose as an energy donor in acetate growing *Acinetobacter calcoaceticus* . Arch Microbiol 144: 62–66.

[96] Lee-Peng FC, Hermodson MA, Kohlhaw GB (1979) Transaminase B from *Escherichia coli*: quaternary structure, amino-terminal sequence, substrate specificity, and absence of a separate valine-alpha-ketoglutarate activity. J Bacteriol 139: 339–345. 378964 10.1128/jb.139.2.339-345.1979 PMC216874

[97] Powell JT, Morrison JF (1978) Role of the *Escherichia coli* aromatic amino acid aminotransferase in leucine biosynthesis. J Bacteriol 136: 1–4. 361681 10.1128/jb.136.1.1-4.1978 PMC218624

[98] Nigro SJ, Hall RM (2011) GI*sul2*, a genomic island carrying the *sul2* sulphonamide resistance gene and the small mobile element CR2 found in the *Enterobacter cloacae* subspecies *cloacae* type strain ATCC 13047 from 1890, *Shigella flexneri* ATCC 700930 from 1954 and *Acinetobacter baumannii* ATCC 17978 from 1951. J Antimicrob Chemother 66: 2175–2176. 21653606 10.1093/jac/dkr230

[99] Tatusov RL, Koonin EV, Lipman DJ (1997) A genomic perspective on protein families. Science 278: 631–637. 9381173 10.1126/science.278.5338.631

[100] Stothard P, Wishart DS (2005) Circular genome visualization and exploration using CGView. Bioinformatics 21: 537–539. 15479716 10.1093/bioinformatics/bti054

[101] Stover BC, Muller KF (2010) TreeGraph 2: combining and visualizing evidence from different phylogenetic analyses. BMC Bioinformatics 11: 7. 20051126 10.1186/1471-2105-11-7 PMC2806359

[102] Carver TJ, Rutherford KM, Berriman M, Rajandream MA, Barrell BG, et al (2005) ACT: the Artemis Comparison Tool. Bioinformatics 21: 3422–3423. 15976072 10.1093/bioinformatics/bti553

[103] Darling AE, Mau B, Perna NT (2010) progressiveMauve: multiple genome alignment with gene gain, loss and rearrangement. PLoS One 5: e11147. 20593022 10.1371/journal.pone.0011147 PMC2892488

