# Supplementary figures and images for: The Complete Genome and Phenome of a Community-Acquired Acinetobacter baumannii
Source: PLoS One. 2013 Mar 19;8(3):e58628. doi: 10.1371/journal.pone.0058628 (PMC3602452; doi:10.1371/journal.pone.0058628)

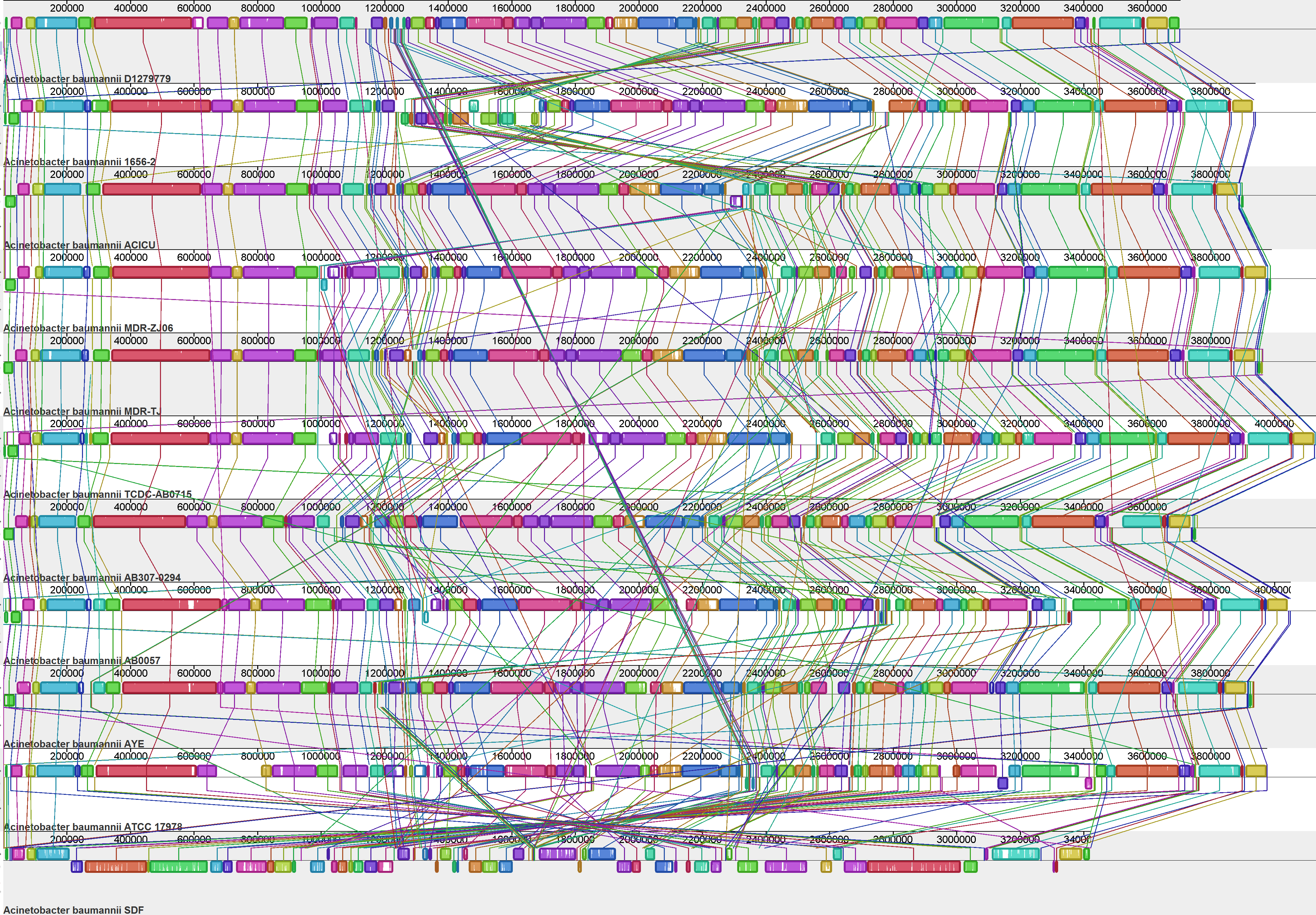

Supplement: Figure S1 — Synteny of Acinetobacter baumannii . Chromosomal alignments of the A. baumannii D1279779 genome against the ten currently complete A. baumannii genomes were generated using progressive MAUVE [103]. Regions of significant synteny between the strains are shown as coloured blocks and unshared regions are seen as white gaps. (PNG) [file pone.0058628.s001.png]

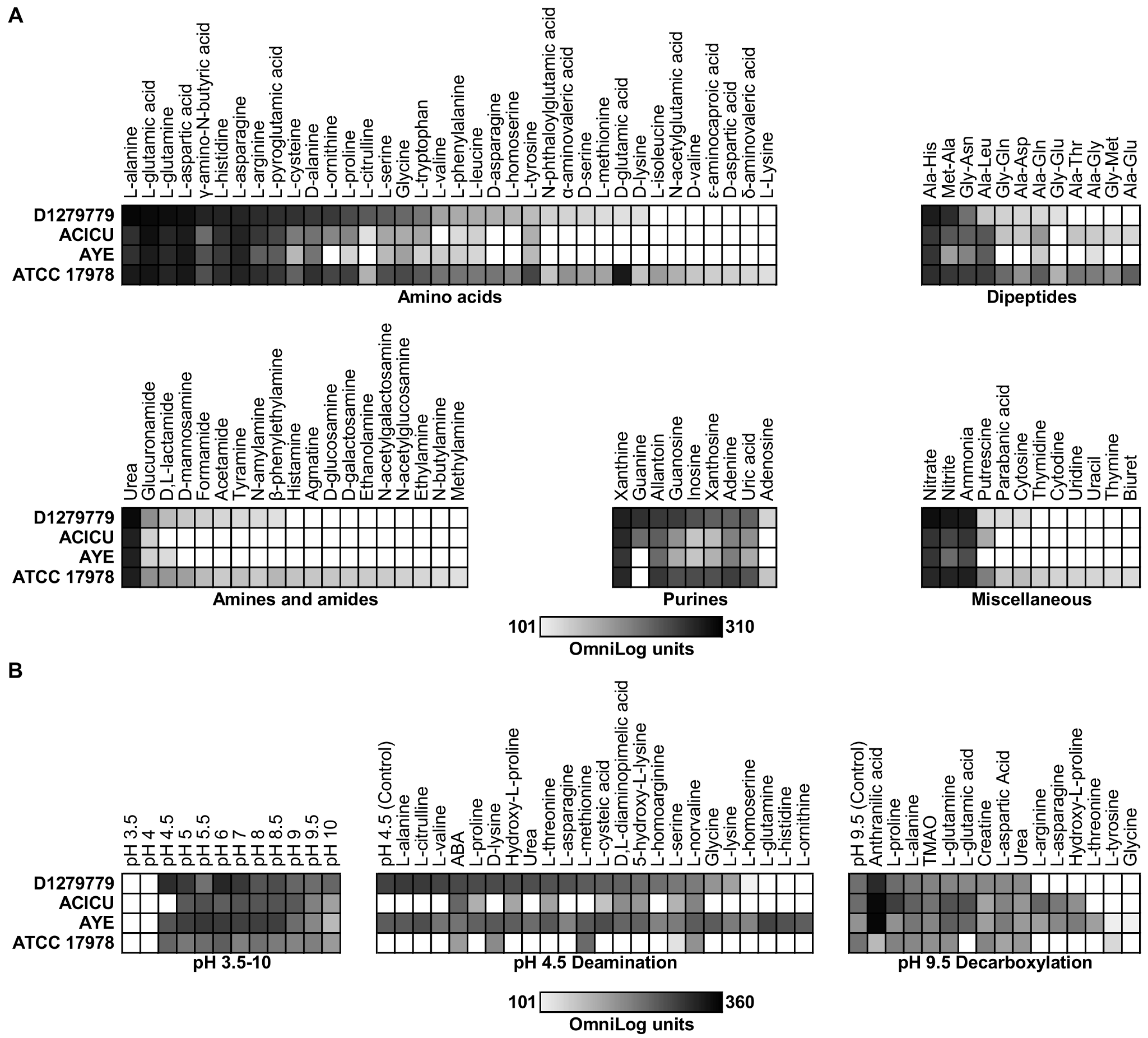

Supplement: Figure S2 — Phenotypic analysis of nitrogen utilisation and pH stress tolerance. Strengths of nitrogen utilisation (A) and the pH tolerance phenotypes (B) of A. baumannii strains D1279779, ACICU, AYE and ATCC 17978 were determined were determined using Biolog Phenotype Microarray plates PM3 and PM10, respectively. The maximal kinetic curve height was expressed as a greyscale ranging from 101 OmniLog units (light grey) to 310 and 360 OmniLog units (black) for nitrogen and pH tolerance phenotypes, respectively. Phenotypes are arranged from strongest to weakest relative to A. baumannii D1279779. Phenotypes <115 OmniLog units for nitrogen phenotypes and <101 OmniLog units for pH tolerance phenotypes were considered negative phenotypes and are represented in white. (TIF) [file pone.0058628.s002.tif]
